# A Fast Na^+^/Ca^2+^-Based Action Potential in a Marine Diatom

**DOI:** 10.1371/journal.pone.0004966

**Published:** 2009-03-23

**Authors:** Alison R. Taylor

**Affiliations:** 1 The Marine Biological Association of the UK, Citadel Hill, Plymouth, United Kingdom; 2 The Department of Biology and Marine Biology, University of North Carolina, Wilmington, North Carolina, United States of America; Temasek Life Sciences Laboratory, Singapore

## Abstract

**Background:**

Electrical impulses in animals play essential roles in co-ordinating an array of physiological functions including movement, secretion, environmental sensing and development. Underpinning many of these electrical signals is a fast Na^+^-based action potential that has been fully characterised only in cells associated with the neuromuscular systems of multicellular animals. Such rapid action potentials are thought to have evolved with the first metazoans, with cnidarians being the earliest representatives. The present study demonstrates that a unicellular protist, the marine diatom *Odontella sinensis*, can also generate a fast Na^+^/Ca^2+^ based action potential that has remarkably similar biophysical and pharmacological properties to invertebrates and vertebrate cardiac and skeletal muscle cells.

**Methodology/Principal Findings:**

The kinetic, ionic and pharmacological properties of the rapid diatom action potential were examined using single electrode current and voltage clamp techniques. Overall, the characteristics of the fast diatom currents most closely resemble those of vertebrate and invertebrate muscle Na^+^/Ca^2+^ currents.

**Conclusions/Significance:**

This is the first demonstration of voltage-activated Na^+^ channels and the capacity to generate fast Na^+^-based action potentials in a unicellular photosynthetic organism. The biophysical and pharmacological characteristics together with the presence of a voltage activated Na^+^/Ca^2+^ channel homologue in the recently sequenced genome of the diatom *Thalassiosira pseudonana*, provides direct evidence supporting the hypothesis that this rapid signalling mechanism arose in ancestral unicellular eukaryotes and has been retained in at least two phylogenetically distant lineages of eukaryotes; opisthokonts and the stramenopiles. The functional role of the fast animal-like action potential in diatoms remains to be elucidated but is likely involved in rapid environmental sensing of these widespread and successful marine protists.

## Introduction

Action potentials are a widespread signalling phenomenon in animals, plants and algae. In animals, the fast Na^+^ based action potential is thought to have evolved along with earliest neuromuscular systems [1]–[3]. In Charophycean green algae (e.g. *Chara* and *Nitella*) and higher plants (Embryophyceae), fundamental features distinguish action potentials characterised from those in animals, including slow kinetics [4]–[6] and the voltage activated ionic conductances that underpin excitability [7]. In contrast, the prevalence and use of membrane excitability during signalling in other photosynthetic lineages such as eukaryotic marine phytoplankton are unknown. Indeed, there have been only a few studies of membrane biophysics in these evolutionary diverse organisms [8]–[10]. Marine diatoms are a particularly successful group of phytoplankton that arose as a result of secondary endosymbiosis of a red algal cell by a heterotrophic eukaryote [11]. The precise origins of the genes in modern heterokont diatoms i.e. either from plastid or nuclear genomes of the ancestral host or red algal symbiont, remains unclear. However, analysis of the *Thalassiosira pseudonana* genome [12], a centric diatom, reveals that approximately 20% of the predicted diatom proteins have significant homology only to known animal proteins, supporting the hypothesis that the heterotrophic host nucleus genome was retained. Moreover, the *T. pseudonana* genome also provides evidence that genes derived from the ancestral heterotrophic host play a key role in the successful adaptation of these photosynthetic organisms to the marine environment [12]. The recent availability of the pennate *Phaeodactylum tricornutum* genome [13] also supports the endosymbiotic origin of diatoms and heterokonts in general. Interestingly, both diatoms and *P. tricornutum* in particular, possess a remarkably high number of genes derived from bacteria, many of which could potentially contribute to novel metabolisms and environmental sensing [13]. The evolutionary origins and ecological importance of marine diatoms therefore led to this study to examine the biophysical properties of the diatom plasma membrane. The work revealed the remarkable finding that the diatom *Odontella sinensis* can generate animal-type action potentials that are unlike other algal and higher plant action potentials.

## Results and Discussion

**Figure 1:**
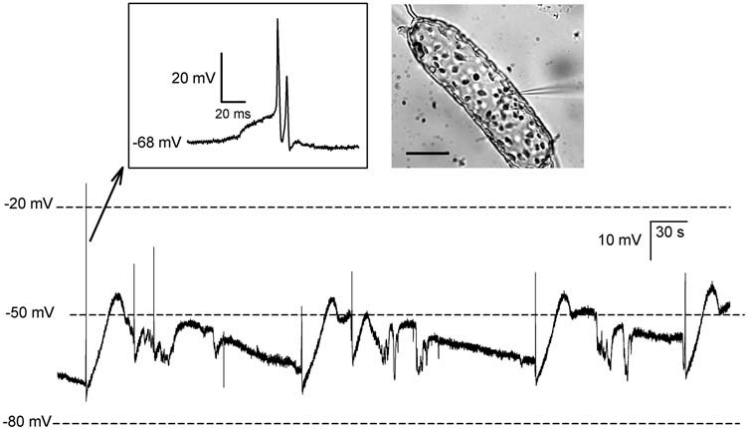
Membrane potential and spontaneous firing action potentials in *Odontella sinensis*. The main trace illustrates a representative free running membrane potential recording in single electrode current clamp mode. Electrodes were inserted through the frustule into the cytoplasmic rich nuclear region of the cell (photomicrograph inset, scale bar 20 µm). The average resting membrane potential was −84 mV (SE±3, n = 12). Over the period of several minutes the membrane potential underwent slow depolarising oscillations preceded by spontaneous fast action potentials (trace inset).

**Figure 2:**
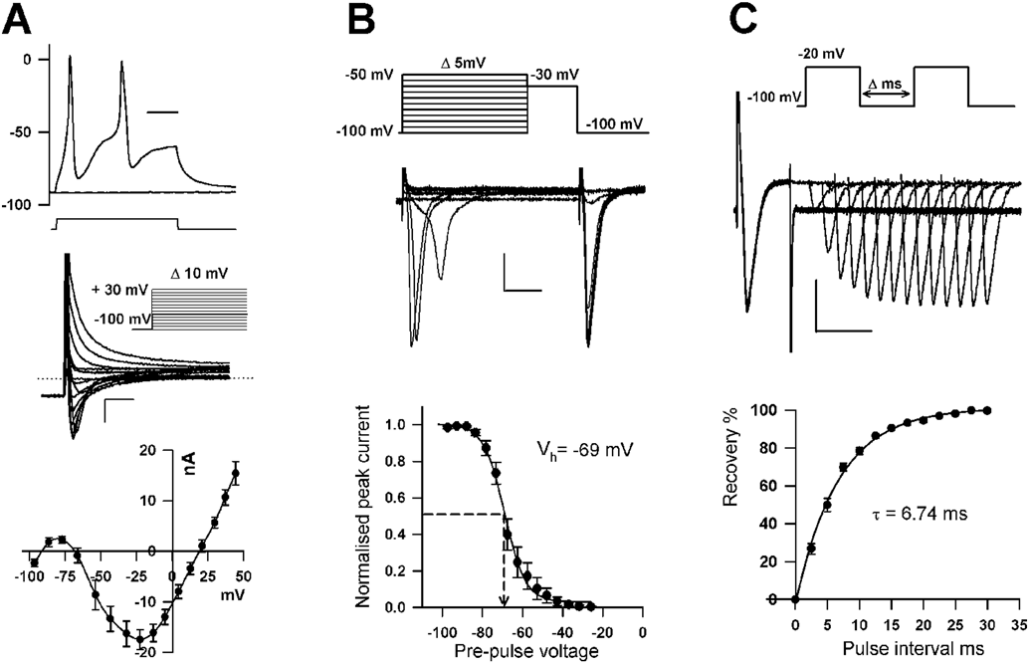
Biophysical characteristics of the *Odontella sinensis* action potential. A) The upper panel shows a current clamp recording illustrating typical action potentials elicited by a brief 1 nA current injection pulse. The average threshold voltage for eliciting and action potential was −68 mV (SE±3, n = 12) with peak depolarisation reaching +21 mV (SE±2, n = 12 ) before the repolarising phase. The time scale bar represents 50 ms. The middle panel shows a representative family of whole cell currents recorded in response to a series of voltage clamp depolarisations from a holding potential of −100 mV to +30 mV (indicated by the inset). Scale bars represent 10 nA and 5 ms and dotted line represents 0 nA. The lower panel consists of an average current voltage curve of the peak inward current elicited by membrane depolarisations. The current peaks at −22.3 mV (SE±1.7, n = 38) and reverses at +20.0 mV (SE±1.9, n = 38). Standard error bars for both current and voltage are indicated. B) Voltage dependent inactivation. The upper panel illustrates a typical experiment where the cell was treated with a progressively depolarised pre-pulse before eliciting a peak inward current with a depolarisation stimulus of −30 mV (protocol indicated by inset). The time scale bar represents 10 ms and the current scale bar represents 10 nA. Capacity transients of the current responses are masked from the traces. Lower panel shows a plot of the average normalised peak current against pre-pulse voltage which is described by a Boltzmann curve (n = 12). The voltage at which half of the channels are inactivated (V_inact_) is −69 mV. C) Recovery from inactivation. The upper panel illustrates a typical experiment where the inward current is elicited with a depolarising voltage clamp pulse followed by a second identical stimulation with varying delay (voltage clamp protocol indicated by inset). The first pulse results in transient inactivation of Na^+^ channels which progressively recover from this state of inactivation as the delay between the first and second pulse increases. The lower panel is a plot of the second evoked peak current expressed as a percentage of the first peak plotted against the time interval (averaged from 16 experiments). The resulting curve is fitted by a single exponential giving a time constant of 6.74 ms for recovery from inactivation. Capacity transients of the current responses to the second stimulus are masked from the traces. The current scale bar represents 10 nA and the time scale bar presents 10 ms.

Spontaneous generation of fast action potentials were frequently recorded on impaling *Odontella sinensis* cells with a sharp intracellular electrode and recording free running membrane potential (Figure 1). Often a secondary slow depolarizing phase was initiated by these spontaneous action potential events, resulting in slow membrane oscillations. Further analysis of the membrane excitability was focussed on the fast action potentials, which could be evoked in 100% of *Odontella sinensis* cells by current injection pulses (Figure 2A). Single electrode voltage clamp recordings showed that the rapid depolarising phase of the action potential is generated by a fast inward current activating at voltages more positive than −65 mV (n = 38). This current peaked at −20 mV and reversed at +20 mV (Figure 2A). The fast action potential current exhibited the features of voltage activation, voltage inactivation and recovery from inactivation that strongly resemble those of animal voltage activated Na^+^ currents (Figure 2B,C). Inactivation is an important characteristic of Na^+^ channels that underlie membrane excitability. Fast activation of Na^+^ currents is followed by rapid voltage –dependent inactivation that facilitates recovery of membrane potential necessary before further action potentials are elicited. Steady state inactivation of the current underlying the diatom action potential was therefore determined using a series of 600 ms voltage clamp pre-pulses immediately prior to activating the action potential current with a voltage clamp step between −30 and −20 mV, where peak inward currents were observed (Figure 2B). The Boltzmann fits to these inactivation curves yielded a V_inact_ of −69 mV (±2, n = 12). The action potential current recovered rapidly from inactivation with a time constant of 6.74 ms (±0.24, n = 16, Figure 2C). These biophysical features are almost identical those of animal Na^+^/Ca^2+^ based action potentials [14], underpinning the shape and speed of action potential impulse by facilitating rapid recovery from the depolarised membrane state.

The recovery phase of animal action potentials is typically dependent on a large but more slowly activating outward K^+^ conductance [15]. In the diatom, the dominant current during the action potential was the fast, but rapidly inactivating inward current. Nevertheless, persistent outward currents were observed at positive potentials after inactivation of the Na^+^ current (e.g. Figure 2). While the inward depolarising current was the focus of the current study, the nature of the outward conductance and to what degree it contributes to membrane repolarisation during the diatom action potential warrants further investigation.

**Figure 3:**
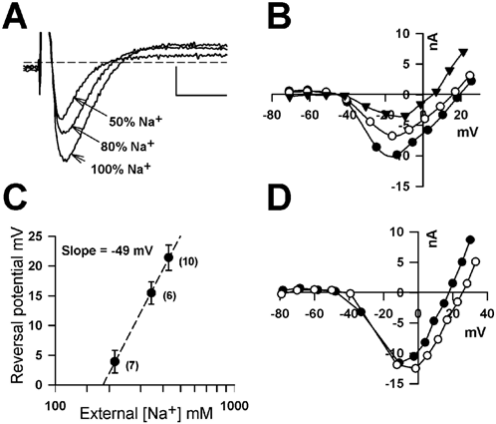
Na^+^ and Ca^2+^ permeation during the diatom action potential. A) The Na^+^ selectivity of the rapid transient current is illustrated by the decline in peak current when Na^+^ ions are substituted with N-methyl D-glucamine. The scale bars represent 5 ms and 5 nA. B) Current-voltage curves for the peak current in the same cell in 100% Na^+^ ASW (filled circles), 80% Na^+^ ASW (open circles) and 50% Na^+^ ASW (filled triangles) illustrate the Na^+^-dependent shift in the reversal potential. C) Plot of average reversal potential against concentration of Na^+^ ions in the external medium reveals a slope of −49 mV which is close to the slope predicted by the Nernst relationship (−58 mV). D) Current voltage curves from the same cell perfused with 10 mM Ca^2+^ ASW (filled circles) and 30 mM Ca^2+^ ASW showing the positive shift in reversal potential. This treatment was repeated for several different cells and revealed an average of +10 mV shift (SE±1.5 mV, n = 10). Standard error bars are indicated.

The depolarising phase of both cardiac and many lower invertebrate fast action potentials is mediated by both Na^+^ and Ca^2+^ influx. The Na^+^ and Ca^2+^-dependency of the dominant inward current underlying the diatom action potential was therefore determined (Figure 3). Replacement of external Na^+^ with either choline or N-Methyl-D-Glucamine caused a negative shift in the reversal potential. The slope of the peak current against external Na^+^ concentration revealed that the inward current is primarily carried by Na^+^ ions (Figure 3C). Significant Ca^2+^ permeability was also indicated by the 12 mV shift in the reversal potential of the peak inward current when changing external Ca^2+^ from 10 to 30 mM (Figure 3D). Thus the diatom action potential, like cardiac and muscle action potentials, not only mediates rapid membrane depolarisation, but also influx of Ca^2+^ ions thereby coupling excitation to intracellular signalling.

**Figure 4:**
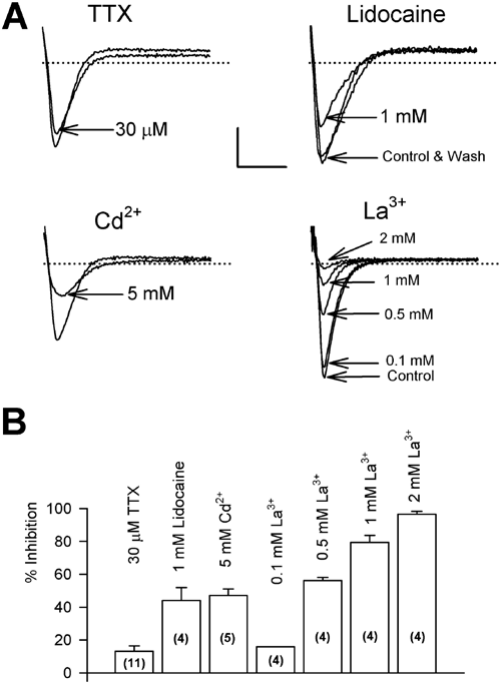
Pharmacology of the diatom Na^+^ current. Peak Na^+^ currents were evoked by a voltage clamp pulse to −20 mV from a holding potential of between −100 mV and −90 mV before perfusion of various pharmacological agents. A) TTX had only a slight impact on the current even at relatively high concentrations and no effect of 1 µM STX was observed (n = 5, data not shown). The Na^+^ current was sensitive to block by lidocaine, Cd^2+^ and La^3+^ ions. In all cases the block was reversible (e.g. lidocaine) on washing out with fresh ASW. Scale bars indicate 10 nA and 5 ms and dotted line represents 0 nA. B) Summary of the effect of various Na^+^ channel antagonists on the peak sodium current in *Odontella sinensis*. Numbers of experiments and standard error bars are indicated for each treatment.

Cardiac-type Na^+^ channels exhibit distinct pharmacological properties when compared to neuronal Na^+^ channels [14]. The pharmacology of the diatom Na^+^ current was therefore examined. The current was insensitive to the potent Na^+^ channel blocker tetrodotoxin (TTX) with only slight block induced with concentrations higher than 5 µM (Figure 4). Not unexpectedly the diatom Na^+^ current was also insensitive to the algal toxin saxitoxin (STX) that blocks at the same site as TTX (Figure 4). Such TTX insensitivity is common among cardiac and invertebrate Na^+^ channels studied to date. This group of TTX insensitive animal Na^+^ channels are however sensitive to lipophyllic blockers [14] and this was also the case with the diatom Na^+^ current, which was significantly blocked by the Na^+^ channel modulating local anaesthetic lidocaine (Figure 4). Cd^2+^ and La^3+^ which are blockers of the TTX-resistant Na^+^ channel [16] were also effective inhibitors of the diatom Na^+^ current (Figure 4).

**Figure 5:**
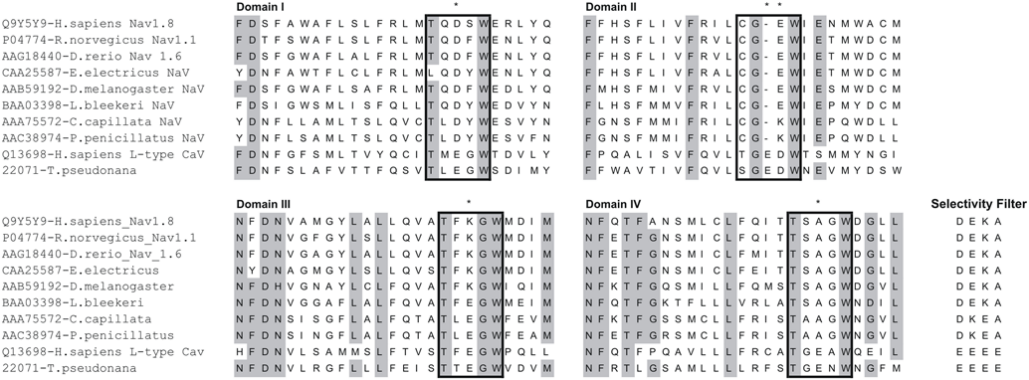
Pore regions and selectivity motifs of animal and diatom voltage activated Na^+^ and Ca^2+^ channels. ClustalW was used to perform multiple sequence alignment of the 4 ‘P’ loop domains of animal and diatom voltage activated Na^+^/Ca^2+^ channels. The selectivity motifs for each pore domain are indicated by boxes with conserved residues conferring selectivity indicated by asterisk. All 4 residues that contribute to the selectivity filter are indicated [34]. Protein sequences were obtained from NCBI and accession numbers are indicated. The diatom sequence for the putative 4 domain voltage activated Na^+^ permeable channel was obtained through a BLAST search of the *Thalassioira pseudonana* genome, Protein ID 2207, http://genome.jgi-psf.org/Thaps3/Thaps3.home.html.

The biophysical and pharmacological properties of the diatom Na^+^ current are remarkably similar to those of invertebrates and of vertebrate cardiac muscle. The question then arises as to the presence of a gene or genes in diatoms that can code functional voltage activated Na^+^ channels with the characteristics observed in this study. The α subunit of animal Na^+^ channels is a large 4-domain membrane spanning protein [17], [18], which may have originated from the single domain bacterial Na^+^ channel [19]. Each of the four domains consists of six transmembrane units designated S1–6. Voltage sensing and gating is achieved by positive charged arginine residues in the S4 units. Voltage dependent inactivation of Na^+^ channels is determined by a highly conserved IMFT motif of the domain III–IV linker of the α subunit [18], [20] and the channel selectivity is determined by the so called ‘P’ loop of S5–S6 linker. Using a range of vertebrate and invertebrate Na^+^ channel amino acid sequences, a BLAST search was conducted on the genome sequence of the related marine diatom *T. pseudonana* [12]. One voltage-activated channel gene with high homology with both the invertebrate Na^+^ channel and vertebrate Na^+^ and Ca^2+^ channel α subunits was found to be present [21]. The predicted amino acid sequence of this gene codes for 4 domains of six membrane spanning units each of which includes both the voltage sensing S4 motifs and the P loops between S5 and S6 as described above. Interestingly, this predicted diatom 4-domain voltage-activated channel exhibits conserved P-loop motif of 4 conserved glutamate residues that result in a selectivity filter that is more alike those described for animal voltage gated Ca^2+^ channels (Figure 5). Overall the putative diatom channel has the predicted structure and function that is consistent with all of the biophysical characteristics described here. This gene and homologues in *O.sinensis* are thus promising candidates for molecular characterisation of the ion channels underpinning diatom membrane excitability and signalling.

Determining a functional role for the action potential in non-motile pelagic diatoms is an important challenge. In plants and large algal cells a limited number of functions for their slow action potentials are proposed, primarily long distance communication associated with mechanosensing and wounding. In the unicellular green alga *Chlamydomonas*, rapid membrane excitability plays a central role in light sensing and swimming behaviour [22]. The pennate diatom *Phaeodactylum tricornutum* also responds rapidly to specific environmental stimuli such as mechanical stress and osmotic shock with increases in cytoplasmic Ca^2+^ concentration [23]. Thus, the fast action potential in diatoms may underlie the initiation of rapid Ca^2+^ based signalling associated with an array on abiotic and biotic stimuli such as shifts in osmotic balance, mechanosensitivity [24] and wound induced activation of chemical defence during grazing by zooplankton [25], [26]. The presence of voltage activated Na^+^ conducting channels in diatoms also raises intriguing questions related to channel blocking toxins in the aquatic environment. Interestingly, the diatom Na^+^ current is insensitive to the peptide STX ruling it out as a novel target for this marine algal toxin. Nevertheless, the sensitivity to lidocaine raises the possibility that the production of some lipophyllic polyether Na^+^ channel toxins by marine dinoflagellates [27] and cyanobacteria [28] could be related to interspecific competition in phytoplankton communities.

While the functional significance of the diatom action potential remains to be fully elucidated, the presence of Na^+^/Ca^2+^ based action potentials in marine diatoms is significant with regard to the evolution of eukaryotic voltage activated Na^+^ channels and the ability to generate fast Na^+^-based electrical impulses. The data presented here provides strong evidence supporting the hypothesis that this signalling mechanism arose before the evolution of the metazoa and has been retained in at least two separate eukaryotic lineages of the tree of life [29]; the photosynthetic heterokont stramenopiles and non-photosynthetic heterotrophic opisthokonts. Evidence of fast Na^+^-based action potentials in other protist groups is currently limited. However, voltage activated Ca^2+^ currents underlie ciliary beating in the alveolate *Paramecium* [30] and Na^+^ permeable conductances have been observed in a heterotrophic dinoflagellate alveolate [31]. Moreover, the marine haptophyte *Coccolithus pelagicus* exhibits membrane excitability that shows remarkably similar kinetics to the Na^+^/Ca^2+^ -based action potential described here [10]. These observations from other representatives of both the chromist and alveolate groups support the hypothesis that the diatom's ability to generate animal-like action potentials was acquired and retained from the ancestral eukaryote protist host. Finally, a Na^+^/Ca^2+^ based action potential in the marine heliozoan *Actinocoryne contractilis* [32], a member of the Rhizaria, further suggests that the ability to utilise fast Na^+^/Ca^2+^ action potentials during environmental sensing and signalling could be widespread among both heterotrophic and autotrophic marine protists.

While further work to examine voltage activated channels and membrane excitability in the chromist, alveolate and protozoan groups is clearly required, the simplest explanation for the presence of voltage activated Na^+^ conducting channels and the capacity to generate fast Na^+^-based action potentials in the marine diatom is that they were acquired and retained from the nuclear genome of the ancestral marine protozoan host and thus arose before the evolution of metazoa and neuromuscular systems.

## Materials and Methods

Individual *Odontella sinensis* cells were isolated from coastal waters near Plymouth, UK, and grown in filtered seawater supplemented with nutrients as previously described [33]. The unialgal cultures were maintained in 100 mL batches at 15°C under 12∶12 day∶night cycle of 150 µmol m^−2^ s^−1^ light. Aliquots of diatom cells were allowed to settle onto the coverslip of a 30 mm Petri dish chamber and perfused with artificial seawater (ASW) consisting of; 450 mM NaCl, 30 mM MgCl_2_, 16 mM MgSO_4_, 8 mM KCl, 10 mM CaCl_2_, 2 mM NaHCO_3_ for a few minutes before commencing electrophysiological recordings. Microelectrodes were fabricated from GC150F glass capillaries (Clark Electromedical; Pangbourne, UK) using a Sutter P47 electrode puller (Sutter Instruments, Petaluma, USA). Electrode tips were coated with beeswax to minimise stray capacitance before filling with 3M KCl or CsCl. Electrodes between 6–10 MΩ were found to enable the best voltage clamp while minimising cell damage. The approximately cyclindical cells (50–70 µm long by 20–40 µM in diameter) were impaled though the silica frustule aided by capacitance over compensation. Single electrode current clamp and voltage clamp experiments were conducted using Clampex software to drive an Axoclamp 2A amplifier, connected to a computer via a Digidata 1200A interface (Axon Instruments, Union City, USA). Clamp speed was typically between 6–12 KHz. Data analysis was carried out offline using Clampfit 8.0 (Axon Instruments).
